# Prevalence of Overweight and Obesity among European Preschool Children: A Systematic Review and Meta-Regression by Food Group Consumption

**DOI:** 10.3390/nu11071698

**Published:** 2019-07-23

**Authors:** Miriam Garrido-Miguel, Andreia Oliveira, Iván Cavero-Redondo, Celia Álvarez-Bueno, Diana P Pozuelo-Carrascosa, Alba Soriano-Cano, Vicente Martínez-Vizcaíno

**Affiliations:** 1 Centro de Estudios Socio-Sanitarios, Universidad de Castilla-La Mancha, 16071 Cuenca, Spain; 2 EPIUnit–Instituto de Saúde Pública da Universidade do Porto (Institute of Public Health, University of Porto), 4050-091 Porto, Portugal; 3 Department of Public Health and Forensic Sciences and Medical Education, Faculty of Medicine, University of Porto, 4200-319 Porto, Portugal; 4 Universidad Politécnica y Artística del Paraguay, 1101 Asunción, Paraguay; 5 Facultad de Ciencias de la Salud, Universidad Autónoma de Chile, 3467987 Talca, Chile

**Keywords:** excess weight, overweight, obesity, pre-school-aged children, Europe, prevalence, food, systematic review

## Abstract

The aim of this review was to estimate the prevalence of overweight and obesity among European children aged 2–7 years from 2006 to 2016 and to analyze these estimations by gender, country, and food group consumption. We searched CINAHL, EMBASE, MEDLINE, and Web of Science databases from their inception until 27 February 2019 including cross-sectional studies and baseline measurements of cohort studies with overweight and obesity defined according to the International Obesity Task Force criteria. Both the inverse-variance fixed-effects method and the DerSimonian and Laird random effects method were used to determinate pooled prevalence estimates and their respective 95% confidence intervals (CIs). A total of 32 studies (*n* = 197,755 children) with data from 27 European countries were included. Overall, the pooled prevalence estimates of overweight/obesity in European children (aged 2–7 years) during the period 2006–2016 was 17.9% (95% CI: 15.8–20.0), and the pooled prevalence estimate of obesity was 5.3% (95% CI: 4.5–6.1). Southern European countries showed the highest prevalence of excess weight. Additional measures to address the obesity epidemic in early life should be established, especially in European countries where the prevalence of excess weight is very high.

## 1. Introduction

Childhood excess weight is a serious public health concern that reaches epidemic proportions in almost all regions of the world [1,2]. According to estimates from the Childhood Obesity Surveillance Initiative (COSI), around 19.3% European children aged six years were overweight/obese in 2010 [3,4]. This estimate will have important public health consequences since overweight children tend to become overweight adults [5].

It is widely known that obesity reduces child health-related quality of life and is associated with several health and social consequences [6,7]. These include social stigmatization, school bullying, poor academic performance, mental health disorders [8,9], as well as an increased risk of certain types of cancer, metabolic syndrome, type 2 diabetes mellitus, osteoarticular diseases, and multiple cardiovascular risk factors [7]. Furthermore, childhood obesity is an independent predictor of cardiovascular events in adult life and overall mortality, in such a way that it has been considered that childhood obesity could pose a threat to life expectancy of the youngest populations [10]. Thus primary prevention of excess weight beginning in childhood or even earlier during pregnancy should be a major public health priority.

Social and lifestyle changes in Europe during the last two decades have affected children’s behavior, through unhealthy eating habits and sedentary lifestyles [7]. In particular, the eating habits in some European countries are involved in a changing process to more westernized dietary habits rich in animal proteins, fats, and with low consumption of complex carbohydrates and fiber [11]. This, together with a decrease in energy expenditure and the rising availability of palatable energy-dense foods, can contribute to the increase of obesity prevalence [12].

Although reports regarding the prevalence of childhood obesity across Europe are frequently published, no one has updated research on the prevalence of excess weight in European children (aged 2–7 years) in the last decade. Additionally, European studies often cover children aged above 7 years, include few European countries and, in some cases, rely on self-reported determinations of weight and height [4,13,14]. The retrieval of information for the latest prevalence in overweight and obesity and food consumption among pre-school children is, therefore, imperative to evaluate the success of policies aimed at reducing excess weight in European children.

This study aimed to estimate the prevalence of overweight and obesity among European children aged 2–7 years from 2006 to 2016 and to analyze these estimates by gender, country, and food group consumption.

## 2. Materials and Methods

**Figure 1:**
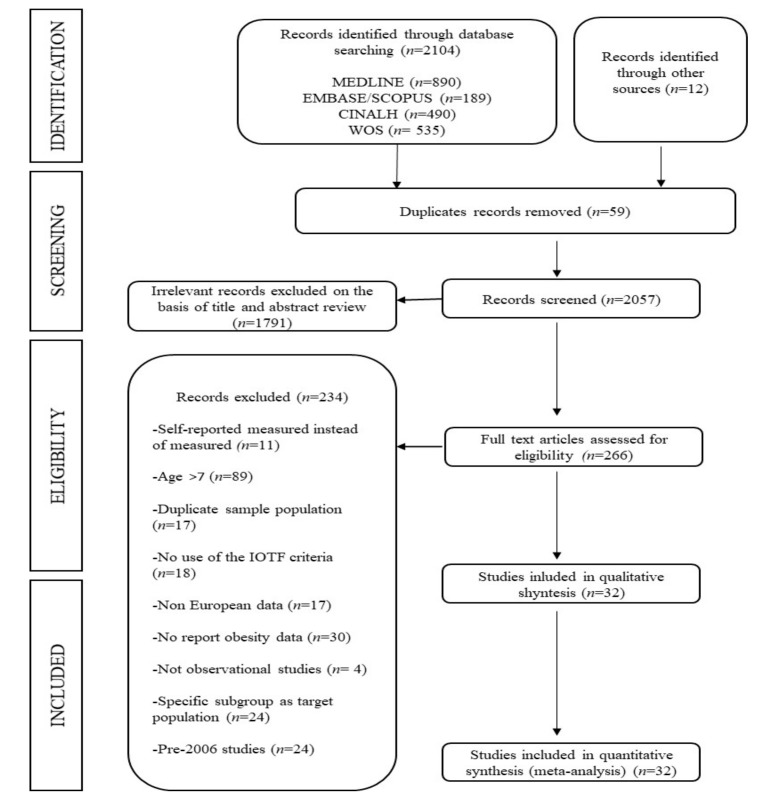
Literature search according to the Preferred Reporting Items for Systematic Reviews and Meta-Analyses (PRISMA) flow chart. Abbreviations: IOTF International Obesity Task Force criteria.

This study was described according to the Preferred Reporting Items for Systematic Reviews and Meta-Analyses (PRISMA) guidelines [15] (Figure 1) and followed the recommendations of the Cochrane Collaboration Handbook [16]. The review was also registered through the International Prospective Register of Systematic Reviews (registration code: CRD42017056924).

### 2.1. Data Sources and Searches

We systematically searched CINAHL, EMBASE, MEDLINE (via PubMed), and Web of Science databases from their inception until 27 February 2019. The search strategy included, combined with Boolean operators, the following terms: (1) population (preschool, infants, toddlers, children, childhood); (2) outcome (obesity, overweight, “body composition”, “body constitution”, “weight status”, anthropometry); (3) study design (prevalence, trend); (4) types of study (observational, cross-sectional, longitudinal); and (5) country (list of European countries). Authors were contacted to obtain missing information when necessary. Additionally, an open search was conducted in health websites to identify obesity estimates not reported in scientific articles (Table S1 in Supplementary Materials).

### 2.2. Study Selection

The criteria for including studies were as follows: (i) studies reporting the population-based prevalence of excess weight (overweight/obesity) or obesity, according to body mass index (BMI) cut-offs proposed by the International Obesity Task Force (IOTF) criteria [17,18]; (ii) study design including cross-sectional studies or baseline measurements of cohort studies with height and weight objectively measured by trained personnel; (iii) studies including individuals aged below 7 years; and (iv) studies published in Portuguese, Spanish, Italian, or English. The exclusion criteria applied were as follow: (i) the sample was a specific subgroup, such as immigrants, or those with a single socioeconomic status; (ii) non-eligible study design, such as review articles, trials, editorials, or comments; and (iii) duplicate documents of the same study.

When more than one study provided data referring to the same sample, we considered the one presenting the results with more detail or providing data for the largest sample size.

The literature search was independently carried out by two authors (M.G.-M. and I.C.-R.), and disagreements were resolved by consultation with a third investigator (V.M.-V.).

### 2.3. Evaluation of Food Consumption

Food consumption information from the European Food Safety Authority (EFSA) European Comprehensive Food Consumption Database were used for the evaluation of food consumption in European children [19,20]. This database collects data from different representative and national dietary surveys carried out in 22 European countries. This food database contained consumption data of 67,000 individuals originating from 33 surveys and covering all groups of ages from infants to the very elderly. In our analysis we used a total of 17 surveys for this evaluation. These studies were selected because they corresponded to the youngest age groups. The 17 European countries included were: Belgium [21], Bulgaria [22], Czech Republic [23], Estonia [24], Finland [25], France [26], Germany [27], Greece [28], Italy [29], Latvia [30], Poland [31], Portugal [32], Romania [19], Spain [33], Sweden [34], The Netherlands [35], and the United Kingdom [36]. We included in our analysis consumption from the following food groups: (1) eggs and egg products, (2) fish (meat), (3) fruits, (4) grains and grain-based products, (5) meat, (6) milk and dairy products, (7) soft drinks, (8) added sugars, and (9) vegetables and vegetable products. Data retrieved were related to chronic food consumption.

### 2.4. Data Extraction and Quality Assessment

Two investigators (M.G.-M. and I.C.-R.) independently revised each published study and collected the following data: (1) country; (2) data collection years; (3) first author’s name; (4) study design; (5) level of representativeness (regional or national); (6) population characteristics (age distribution and sample size); and (7) prevalence of overweight/obesity and obesity based on the IOTF definition criteria by sex [17,18] (Table S2 in Supplementary Materials).

The Joanna Briggs Institute (JBI) tool [37] was used to assess the risk of bias in the prevalence studies. This tool consists of a rating list with ten criteria, which can be assessed as ‘yes’ (=1); ‘no’ (=0), ‘not applicable’ (=NA) or ‘unclear’ (=?); thus, the score for each study ranged from 0 to 10. Depending on this score, we rated each study as low-risk (7–10), moderate-risk (4–6), or high-risk of bias (1–3) (Table S3 in Supplementary Materials).

### 2.5. Statistical Analysis

The Mantel–Haenszel fixed-effects method [38] was used to compute the point prevalence estimates whenever there was no evidence of heterogeneity; otherwise, the DerSimonian and Laird random-effects method was used [39]. Study heterogeneity was evaluated by using the *I*^2^ statistic [40], and the following values of *I*^2^ were used for the interpretation of heterogeneity levels: 0% to 40%, without heterogeneity; 30% to 60%, moderate heterogeneity; 50% to 90%, substantial heterogeneity; and 75% to 100%, considerable heterogeneity. The corresponding *p* values were also taken into account [41]. We used the Mantel–Haenszel fixed-effects method when *I*^2^ was < 50%.

The subgroup analyses were performed according to gender and country. Additionally, a meta-regression analysis was used to assess the relationship between overweight and obesity prevalence estimates and each of the following main food groups: eggs and egg products, fish, fruits, grains and grain-based products, meat, milk and dairy products, and soft drinks and sugars collected from European surveys [19].

The significance value of the pooled effect size was estimated based on the 95% CI. Statistical analyses were performed using STATA SE software, version 15 (StataCorp, College Station, TX, USA).

## 3. Results

### 3.1. Study Selection and Characteristics

The PRISMA flow diagram is presented in Figure 1. From the 2104 full-text articles identified, only 32 studies met the inclusion criteria [3,4,42,43,44,45,46,47,48,49,50,51,52,53,54,55,56,57,58,59,60,61,62,63,64,65,66,67,68,69,70,71] and were included in the systematic review. Of these, four studies displayed data for several European countries [3,4,42,43]. Studies were conducted in 27 European countries: Belgium (3 reports), Bulgaria (1), Cyprus (2), Czech Republic (1), Estonia (1), Finland (1), France (2), Germany (1), Greece (3), Hungary (1), Ireland (2), Italy (3), Latvia (2), Lithuania (1), Malta (1), Poland (4), Portugal (5), Romania (1), Serbia (1), Slovenia (5), Spain (5), Sweden (2), Switzerland (2), The Netherlands (2), Turkey (1), the United Kingdom (1), and Yugoslav Republic of Macedonia (1). A total of 193,755 children were included in this systematic review. These studies were published between 2006 and 2016, included sample sizes ranging from 128 to 52,647 participants with ages ranging from 2 to 7 years old (Table S2 in Supplementary Materials).

### 3.2. Risk of Bias

After evaluation of the risk of bias by the JBI tool [37], 12.5% of the studies were categorized as having a moderate risk of bias, and 87.5% as low risk. When considering the individual domains of the scale, in 81.25% of the studies, the measurement of weight and height was described in detail and met the criteria for reliable measurement (Table S3 in Supplementary Materials).

### 3.3. Data Synthesis

**Figure 2:**
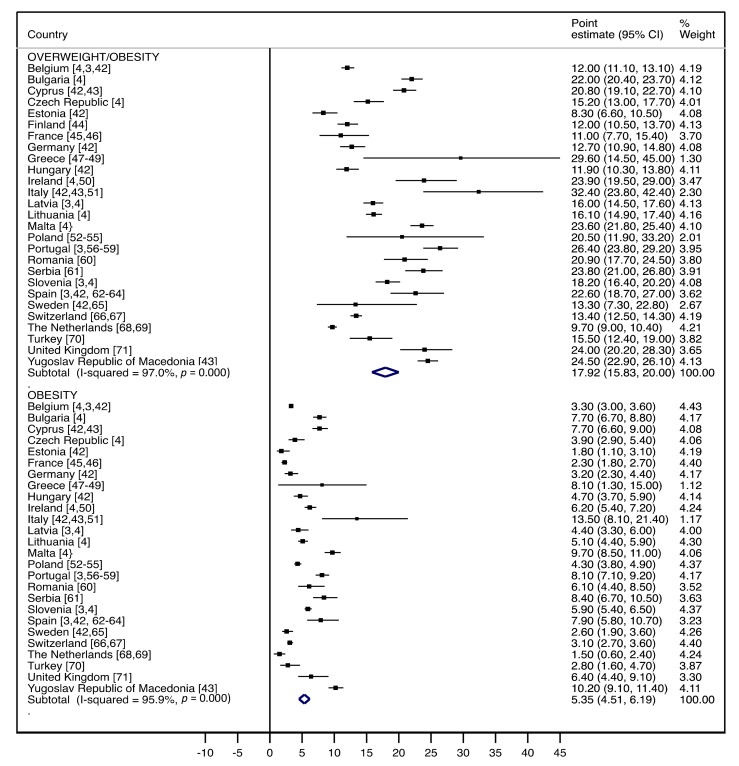
Forest plot of the pooled prevalence means of overweight/obesity, and obesity based on the International Obesity Task Force (IOTF) cutoffs, in European children (aged 2–7 years) from 2006 to 2016.

**Figure 3:**
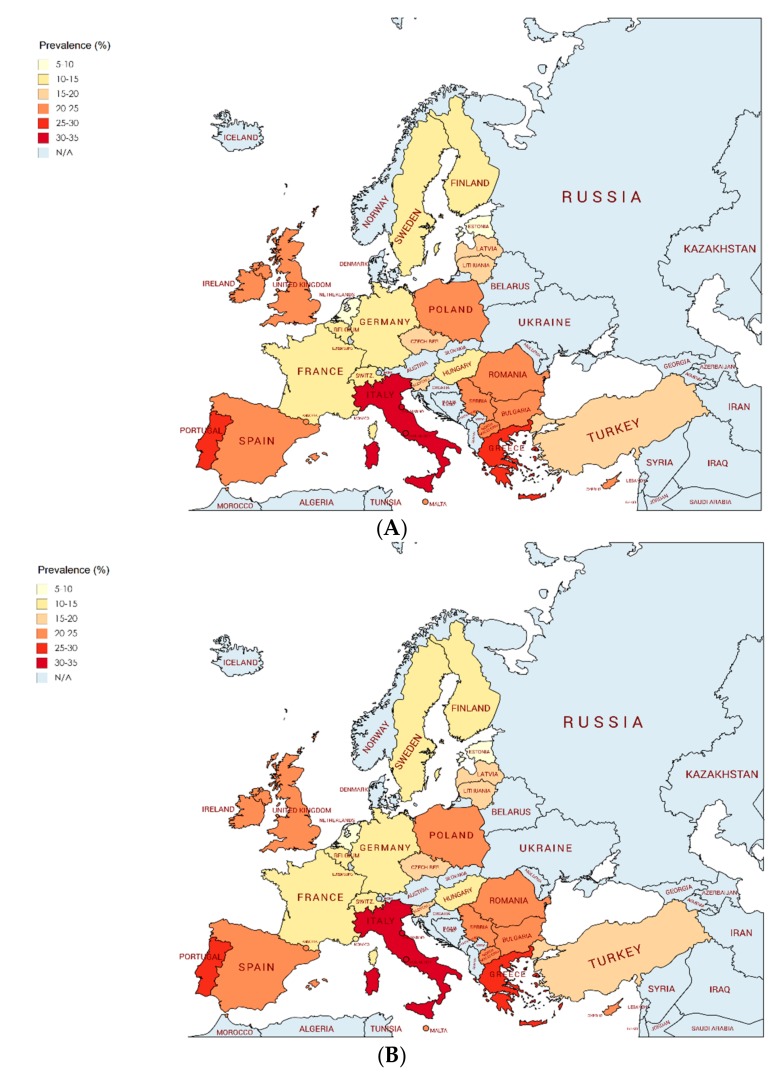
Spatial distribution of the prevalence of overweight/obesity (**A**) and obesity (**B**), based on the IOTF cutoffs, in European children (aged 2–7 years) from 2006 to 2016. Abbreviations: N/A not available.

Figure 2 and Figure 3 shows the prevalence of overweight/obesity and obesity in children aged 2 to 7 years for 27 European countries from 2006 to 2016, using IOTF definition criteria [17,18].

Overall, the pooled prevalence estimates of overweight/obesity in Europe was 17.9% (95% CI: 15.8–20.0). Lower prevalence estimates of overweight/obesity were observed in Estonia (8.3%; 95% CI: 6.6–10.5), France (11.0%; 95% CI: 7.7–15.4), and The Netherlands (13.4%; 95% CI: 12.5–14.3), and higher prevalence estimates were in Italy (32.4%; 95% CI: 23.8–42.4), Greece (29.6%; 95% CI: 14.5–45.0), and Portugal (26.4%; 95% CI: 23.8–29.2).

Overall, the pooled prevalence estimates of obesity in Europe was 5.3% (95% CI: 4.5–6.1). Lower estimates were reported in The Netherlands (1.5%; 95% CI: 0.6–2.4), Estonia (1.8%; 95% CI: 1.1–3.1), and France (2.3%; 95% CI: 1.8–2.7), and higher estimates were seen in Italy (13.5%; 95% CI: 8.1–21.4), Yugoslav Republic of Macedonia (10.2%; 95% CI: 9.1–11.4), and Malta (9.7%; 95% CI: 8.5–11.0).

Girls presented a higher pooled prevalence estimate of overweight/obesity and obesity than boys in most European countries (except in the Czech Republic, Germany, and Serbia). (See Table S4 in Supplementary Materials for sex-specific prevalence in each country).

### 3.4. Meta-Regression

**Figure 4:**
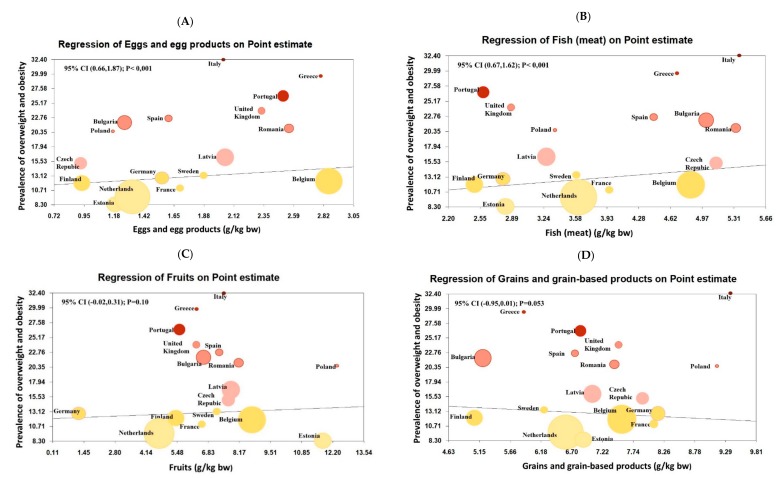

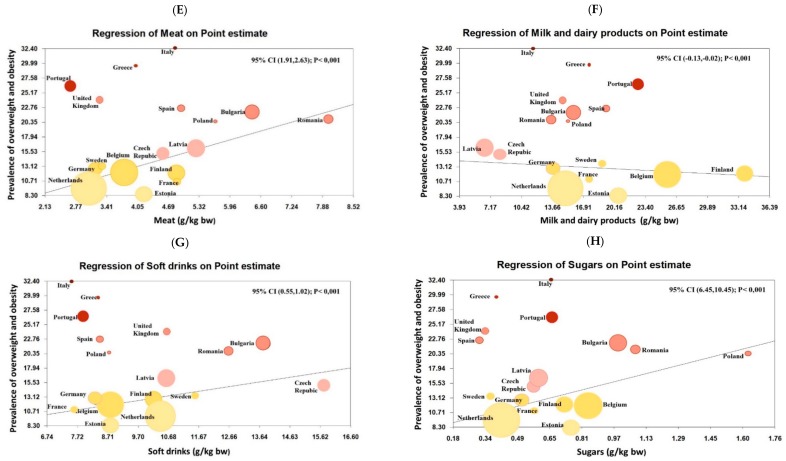

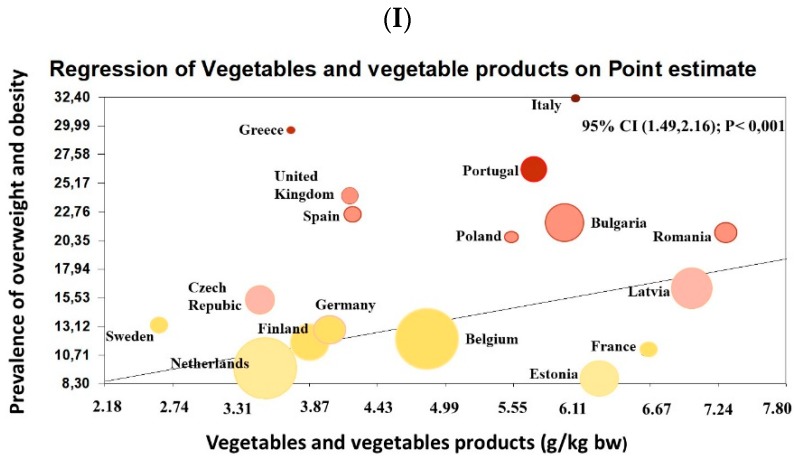
Meta-regression analyses. Plots show the point estimates of the prevalence of overweight/obesity of different countries in Europe during the period (2006–2016) according to different food groups provided by the food consumption data from the European Food Safety Authority (EFSA) (mean quantity in g/kg of body weight). Abbreviations: bw: body weight; g/kg: grams per kilogram; CI: confidence intervals.

Meta-regression models (Figure 4) showed a positive significant association between the pooled prevalence estimate of overweight/obesity with consumption from the following food groups: (**A**) eggs and egg products (coefficient: 1.2; 95% CI: 0.6–1.8), (**B**) fish (meat) (coefficient: 1.1; 95% CI: 0.6–1.6), (**E**) meat (coefficient: 2.2; 95% CI: 2.4–5.4), (**G**) soft drinks (coefficient 0.7; 95% CI: 0.5–1.0), (**H**) added sugars (coefficient: 8.45; 95% CI: 6.4–10.4), and (**I**) vegetables and vegetable products (coefficient: 1.8; 95% CI: 1.4–2.1). Otherwise, negative associations were found with (**F**) milk and dairy products (coefficient: −0.07; 95% CI: −0.1, −0.02). No significant differences were found with (**C**) fruits (coefficient: 0.14; 95% CI: −0.02, 0.3) and (**D**) grains and grain-based products (coefficient: −0.47; 95% CI: −0.9, 0.007). All food groups are described as mean quantity in grams per kilogram (g/kg) of body weight for each European country.

## 4. Discussion

The present study provides a complete picture on the prevalence of overweight and obesity among European children aged 2–7 years during a 10-year period (2006 to 2016) using a consistent, systematic, and transparent methodology. About 17.9% of children aged 2–7 years were identified with overweight or obesity, and 5.3% of children with obesity according to the IOTF definition criteria [17,18]. The prevalence of overweight and obesity is heterogeneously distributed across Europe, as indicated by the important differences between European countries. Thus, southern European countries showed the highest prevalence of overweight and obesity. In this sense, the highest prevalence estimates were observed in Italy (32.4%; 95% CI: 23.8–42.4), Greece (29.6%; 95% CI: 14.5–45.0), and Portugal (26.4%; 95% CI: 23.8–29.2). Otherwise, the lowest prevalence estimates of overweight and obesity were found in Estonia (8.3%; 95% CI: 6.6–10.5), France (11.0%; 95% CI: 7.7–15.4), and The Netherlands (13.4%; 95% CI: 12.5–14.3).

Our study is in line with recent reports [1,9] that described that the prevalence of childhood excess weight in European countries is still very high, especially in countries belonging to the Mediterranean Sea. It is also relevant to take into account that our study also shows a considerable prevalence of overweight and obesity in children (aged 2–7 years) in countries such as the United Kingdom and Ireland (24% and 23.9%, respectively). An important prevalence was also observed in Eastern Europe showing rates between 21 and 24%. The very high prevalence of childhood obesity in some European countries could be somewhat explained by the gradual modification from the healthy traditional diets to a more westernized diet rich in animal proteins, fats, and sugar foods, and poor in complex carbohydrates and fiber [11]. This, together with an increase in sedentary behaviors and a decrease in physical activity [9], probably contributes decisively to the increase in the prevalence of obesity in the last two decades.

Excess weight in childhood is still increasing in some countries [1,2,3,4], and this is paralleled by an important change and westernization of dietary patterns due to socioeconomic, cultural, geography, affective, and marketing factors [9]. This study provides an overall picture of the prevalence of overweight and obesity, according to consumption of different food groups, provided by the EFSA database from different surveys of Europe, using standardized methodologies for the collection of dietary data [19,20]. In our meta-regression analyses, the consumption of animal proteins, and sugars was positively associated with excess weight, whereas the consumption of milk and dairy products was inversely associated with overweight and obesity. Although it represents a very crude ecological analyses, these findings support this relationship. The ecological design by itself prevents us from establishing causal inferences, but it clearly shows a trend and helps to understand how food consumption is distributed in different European countries. Our results show a marked difference in Eastern Europe countries by the higher mean intakes of sugars, soft drinks, and animal proteins, and the lower consumption of whole grain cereals, fruits, and milk. It is explained because numerous authors have suggested that children living in middle and low-income countries are more vulnerable to developing obesity, probably due to poorer eating habits, but mostly because of the increase in the cost of healthier diets [72].

In our study, girls showed a higher prevalence of excess weight than boys in most European countries; this could be explained by numerous factors related with the hormonal biology, as well as environmental and behavioral factors that predispose girls to excessive weight gain throughout life [73,74].

Childhood is a critical period characterized by continuous development, body growth, and physical changes, and the onset of habits that will possibly continue at more advanced ages [9]. In this sense, a high prevalence of excess weight or unhealthy habits in young children is also indicating an increased risk for even higher rates of obesity in later ages, in the near future, exceeding those currently reported. For these reason, additional measures to address the childhood obesity epidemic should be established at an early life, especially in European countries where the prevalence of excess weight is high.

### Limitations

Regarding this review, there are numerous limitations that should be taken into account when interpreting these results. First, only studies that defined overweight and obesity by the IOTF criteria [17,18] were included, which limited the comparability with other studies. We decided to use the IOTF criteria because these cut-offs for children are representative of the entire world’s population, and it allowed us to make better comparability during the period 2006–2016 in different European countries than other international criteria, such as those from the Centers for Disease Control and Prevention [75] or the World Health Organization [76]. Second, studies using the two versions of these IOTF cut-offs were included [17,18], and although differences in the cut-offs were minor, the estimates can be biased. Third, not all studies ensure representative samples of the population and our results should be interpreted with caution. Nonetheless, a quality assessment was performed and none of the studies had a high risk of bias. Fourth, measured data on weight and height were scarcer in some European countries, which posed a threat to the validity of their estimates. Other measures of adiposity distribution, such as waist-to-height ratio, should be also explored as they substantially increase the risk of metabolic complications in the future. Fifth, although we used a meta-regression with EFSA data, we performed this statistical testing only in order to obtain a crude picture of the trend of some food groups in the prevalence of excess weight in different European countries. Sixth, differences in sample characteristics, location at the geographical level, and differences in the quality of included studies could increase the heterogeneity between the studies, which might decrease the quality of evidence in prevalence estimates reported.

## 5. Conclusions

In conclusion, the prevalence of overweight and obesity is still high across Europe among children aged 2–7 years, particularly in Southern countries like Italy and Greece. Current country-specific policies and interventions are reinforced to curb the excess weight found at early ages, especially in countries where the obesity prevalence is still high.

## Supplementary Materials

The following are available online at https://www.mdpi.com/2072-6643/11/7/1698/s1: Table S1: Search strategy for Medline; Table S2: Characteristics of studies included in the systematic review (*n* = 32); Table S3: Quality assessment of prevalence studies included in the systematic review following the Joanna Briggs Institute (JBI) tool; Table S4: Point estimates and 95% confidence intervals for the prevalence of childhood overweight (OW) and obesity (OB) among European children (aged 2–7 years) using the IOTF definition criteria.
