# Supplementary material for: Prevalence of Overweight and Obesity among European Preschool Children: A Systematic Review and Meta-Regression by Food Group Consumption
Source: Nutrients. 2019 Jul 23;11(7):1698. doi: 10.3390/nu11071698 (PMC6682909; doi:10.3390/nu11071698)
Supplement: Supplementary file 1 [file nutrients-11-01698-s001.pdf]

Table S1. Search strategy for Medline.

	Search terms
**1. Population**	(Preschool OR Infants OR Toddlers OR Children OR Childhood)
**2. Outcome**	(Obesity OR Overweight OR "Body composition" OR "Body constitution" OR "Weight status" OR Anthropometr *)
**3. Study design**	(Prevalence OR Trend)
**4. Types of studies**	(Observat * OR "Cross-sectional" OR Longitudinal NOT (Review OR Intervention OR Experimental))
**5. Location**	(Russia OR Germany OR Turkey OR France OR "United Kingdom" OR Italy OR Spain OR Ukraine OR Poland OR Romania OR Kazakhstan OR Netherlands OR Belgium OR Greece OR Czech Republic OR Portugal OR Sweden OR Hungary OR Azerbaijan OR Belarus OR Austria OR Switzerland OR Bulgaria OR Serbia OR Denmark OR Finland OR Slovakia OR Norway OR Ireland OR Croatia OR Bosnia and Herzegovina OR Georgia OR Moldova OR Armenia OR Lithuania OR Albania OR Macedonia OR Slovenia OR Latvia OR Kosovo OR Estonia OR Cyprus OR Montenegro OR Luxembourg OR Malta OR Iceland OR Andorra OR Liechtenstein OR Monaco OR "San Marino" OR "Vatican city" OR Europe * [title])
**1 AND 2 AND 3 AND 4 AND 5**	
Truncation symbol: * = all possible word endings included.	

Table S2. Characteristics of studies included in the systematic review (*n* = 32).

Country	Data Collection Years	First Author	Design. Cole criteria	National or Regional Data	Age Range	Sample Size			Prevalence of Overweight and Obesity (%)			Prevalence of Obesity (%)		
						All	girls	boys	All	girls	boys	All	girls	boys
Belgium	2007–2008	Wijnhoven TMA [1] COSI Round 1	CS. Cole, 2000	Regional (Flanders)	6	49536	24465	25071	13.0	14.9	11.2	3.3	3.8	2.9
Belgium	2007–2008	Ahrens W [2] IDEFICS 2008	Cohort (Baseline data T0). Cole, 2012	Regional (Flanders)	3–6.99	1349	659	690	7.7	8.8	6.7	1.9	2.3	1.6
Belgium	2009–2010	Wijnhoven TMA [3] COSI Round 2	CS. Cole, 2000	Regional (Flanders)	6	52647	26105	26542	12.9	15.1	10.8	3.5	4.2	2.8
Bulgaria	2007–2008	Wijnhoven TMA [1] COSI Round 1	CS. Cole, 2000	National	7	2488	1230	1258	22.0	24.1	20.1	7.7	9.0	6.6
Cyprus	2007–2008	Ahrens W [2] IDEFICS 2008	Cohort (Baseline data T0). Cole, 2012	Regional	3–6.99	1565	767	798	21.0	22.4	19.7	7.7	8.5	7.0
Cyprus	2013–2014	Ahrens W [4] I FAMILY 2013	Cohort. Cole, 2012	Regional	3–6.99	322	148	174	19.9	21.7	18.4	8.1	6.8	9.2
Czech Republic	2007–2008	Wijnhoven TMA [1] COSI Round 1	CS. Cole, 2000	National	7	915	470	445	15.2	14.7	15.7	3.9	4.0	3.8
Estonia	2007–2008	Ahrens W [2] IDEFICS 2008	Cohort (Baseline data T_0). Cole, 2012	Regional	3–6.99	769	376	393	8.3	9.3	7.6	1.8	2.4	1.3
Finland	2007–2009	Lehto R [5]. LATE project	CS. Cole, 2000	Regional (Turku and Kainuu)	3 and 5	1681	N/A	N/A	12.0	N/A	N/A	N/A	N/A	N/A
France	2006–2007	Lioret S [6]	CS. Cole, 2000	National	3–6	228	N/A	N/A	13.6	N/A	N/A	3.0	N/A	N/A
France	2007–2008	Thibault H [7]	CS. Cole, 2000	Regional (Aquitaine region)	5–7	4048	N/A	N/A	9.5	N/A	N/A	2.3	N/A	N/A
Germany	2007–2008	Ahrens W [2] IDEFICS 2008	Cohort (Baseline data T0). Cole, 2012	Regional	3–6.99	1164	558	606	12.7	10.9	14.5	3.2	4.1	2.5
Greece	2006–2007	Smpokos EA [8]	CS. Cole, 2000	Regional (Crete)	5.7–7.8	361	162	199	34.1	34.6	33.7	N/A	N/A	N/A
**Country**	**Data Collection Years**	**First Author**	**Design. Cole criteria**	**National or regional data**	**Age range (years)**	**Sample size**			**Prevalence of overweight and obesity (%)**			**Prevalence of obesity (%)**		
						**All**	**girls**	**boys**	**All**	**girls**	**boys**	**All**	**girls**	**boys**
Greece	2009	Kleanthous K [9] West Attica Growth	Cohort. Cole, 2000	National	67	286	131	155	43.4	44.2	42.6	19.2	21.4	17.5

		Study												
Greece	2013	Pikramenou V [10]	CS. Cole, 2000	Regional (Thessaloniki)	2.5–6.5	2180	1007	1173	16.0	17.4	14.7	3.2	3.2	3.3
Hungary	2007–2008	Ahrens W [2] IDEFICS 2008	Cohort (Baseline data T_0). Cole, 2012	Regional	3–6.99	1350	678	672	11.9	13.0	10.9	4.7	5.8	3.6
Ireland	2007	Barron C [11]	CS. Cole, 2000	Regional (Co Kildare)	4–6	323	147	176	26.9	27.2	26.7	8.0	9.5	6.8
Ireland	2007–2008	Wijnhoven TMA [1] COSI Round 1	CS. Cole, 2000	National	7	2409	1266	1143	21.9	22.8	21.1	5.9	5.6	6.3
Italy	2007–2008	Ahrens W [2] IDEFICS 2008	Cohort (Baseline data T_0) Cole, 2012	Regional	3–6.99	1363	631	732	36.5	38.3	34.8	16.6	16.3	16.9
Italy	2013	Toselli S [12]. SoNIA project	CS. Cole, 2000	Regional (Emilia-Romagna)	5–6	2337	1152	1185	25.2	29.0	21.6	8.9	9.3	8.6
Italy	2013–2014	Ahrens W [4] I FAMILY 2013	Cohort. Cole, 2012	Regional	3–6.99	131	64	67	37.4	42.1	32.8	16.8	17.2	16.4
Latvia	2007–2008	Wijnhoven TMA [1] COSI Round 1	CS. Cole, 2000	National	7	3249	1651	1598	15.2	15.1	15.3	3.8	3.1	4.5
Latvia	2009–2010	Wijnhoven TMA [3] COSI Round 2	CS. Cole, 2012	National	7	2838	1457	1381	16.8	17.7	15.8	5.2	5.3	5.3
Lithuania	2007–2008	Wijnhoven TMA [1] COSI Round 1	CS. Cole, 2000	National	7	3309	1649	1660	16.1	16.2	16.1	5.1	5.1	5.1

Table S3. Quality assessment of prevalence studies included in the systematic review following the Joanna Briggs Institute (JBI) tool.

First author	Was the sample representative of the target population?	Were study participants recruited in an appropriate way?	Was the sample size adequate?	Were the study subjects and the setting described in detail?	Was the data analysis conducted with sufficient coverage of the identified sample?	Were objective, standard criteria used for the measurement of the condition?	Was the condition measured reliably?	Was there appropriate statistical analysis?	Are all important confounding factors/subgroups/differences identified and accounted for?	Were subpopulations identified using objective criteria?
Wijnhoven TMA et al [1]	YES	YES	YES	YES	YES	YES	YES	YES	YES	YES
Ahrens W et al [2]	NO	YES	YES	UNCLEAR	UNCLEAR	YES	YES	YES	YES	YES
Wijnhoven TMA et al [3]	YES	YES	YES	YES	YES	YES	YES	YES	YES	YES
Lehto R et al [5]	UNCLEAR	YES	YES	YES	YES	YES	YES	YES	YES	YES
Lioret S et al [6]	YES	YES	YES	YES	YES	UNCLEAR	UNCLEAR	YES	YES	YES
Thibault H et al [7]	YES	YES	YES	UNCLEAR	YES	UNCLEAR	UNCLEAR	YES	YES	YES
Smpokos EA et al [8]	YES	YES	YES	YES	YES	YES	YES	YES	YES	YES
Kleanthous K et al [9]	UNCLEAR	UNCLEAR	YES	YES	YES	YES	YES	YES	UNCLEAR	YES
Pikramenou V et al [10]	NO	UNCLEAR	YES	UNCLEAR	YES	UNCLEAR	UNCLEAR	YES	N/A	N/A

Barron C et al [11]	UNCLEAR	UNCLAR	YES	YES	YES	YES	YES	YES	YES	YES
Toselli S et al [12]	NO	YES	YES	UNCLEAR	YES	YES	YES	YES	UNCLEAR	UNCLEAR
Bac A et al [13]	NO	UNCLEAR	YES	UNCLEAR	YES	YES	YES	YES	N/A	N/A
Kowal M et al [14]	YES	YES	YES	YES	YES	YES	YES	YES	N/A	N/A
Kułaga Z et al [15]	YES	YES	YES	UNCLEAR	YES	YES	YES	YES	N/A	N/A
Merkiel S et al [16]	YES	YES	YES	YES	YES	YES	YES	YES	N/A	N/A
Bingham D et al [17]	YES	YES	YES	YES	YES	YES	YES	YES	YES	YES
Vale S et al [18]	YES	YES	YES	UNCLEAR	YES	YES	YES	YES	YES	YES
COSI Round 3 [19]	YES	YES	YES	YES	YES	YES	YES	YES	YES	YES
Silva-Santos S et al [20]	YES	YES	YES	YES	YES	YES	YES	YES	YES	YES
Barbu CG et al [21]	YES	YES	YES	YES	YES	UNCLEAR	UNCLEAR	YES	YES	YES
Djordjic V et al [22]	YES	YES	YES	YES	YES	YES	YES	YES	UNCLEAR	UNCLEAR
García García E et al [23]	YES	YES	YES	UNCLEAR	YES	YES	YES	YES	YES	YES
González García A et al [24]	YES	YES	YES	UNCLEAR	YES	YES	YES	YES	YES	YES
Gómez Santos SF et al [25]	NO	YES	YES	UNCLEAR	YES	YES	UNCLEAR	YES	N/A	N/A
Garmy Pet al [26]	NO	YES	YES	YES	YES	UNCLEAR	UNCLEAR	YES	YES	YES
Jeannot E et al [27]	YES	YES	YES	UNCLEAR	YES	YES	YES	YES	N/A	N/A
Jeannot E et al [28]	YES	YES	YES	UNCLEAR	YES	YES	YES	YES	N/A	N/A
de Wilde JA et al [29]	YES	YES	YES	UNCLEAR	YES	UNCLEAR	YES	YES	YES	YES
Schönbeck Y et al [30]	YES	YES	YES	YES	YES	YES	YES	YES	N/A	N/A
Senol V et al [31]	YES	YES	YES	YES	YES	YES	YES	YES	YES	YES
Basterfield et al [32]	YES	YES	YES	UNCLEAR	YES	YES	YES	YES	YES	YES

Abbreviation: NA: Not applicable.

Table S4. Point estimates and 95% confidence intervals for the prevalence of childhood overweight (OW) and obesity (OB) among European children (aged 2-7 years) using the IOTF definition criteria.

2006–2016									
	All			Girls			Boys		
Country	Number of studies	I^2	Pooled estimated (95 % CI)	Number of studies	I^2	Pooled estimated (95 % CI)	Number of studies	I^2	Pooled estimated (95 % CI)
**Belgium**									
OW/OB	3	93.7	12.0 (11.1–13.1)	3	89.7	14.2 (13.1–15.5)	3	86.6	10.5 (9.7–11.5)
OB	3	82.9	3.3 (3.0–3.6)	3	80.5	3.9 (3.4–4.4)	3	52.9	2.8 (2.7–3.0)
**Bulgaria**									
OW/OB	1	–	22.0 (20.4–23.7)	1	-	24.1 (21.8–26.5)	1	-	20.1 (18.0–22.4)
OB	1	-	7.7 (6.7–8.8)	1	-	9.0 (7.5–10.8)	1	-	6.6 (5.4–8.1)
**Cyprus**									
OW/OB	2	0.0	20.8 (19.1–22.7)	2	0.0	22.3 (19.7–25.1)	2	0.0	19.4 (17.1–22.1)
OB	2	0.0	7.7 (6.6–9.0)	2	0.0	8.2 (6.6–10.2)	2	0.0	7.4 (6.0–9.3)
**Czech Republic**									
OW/OB	1	-	15.2 (13.0–17.7)	1	-	14.7 (11.8–18.2)	1	-	15.7 (12.6–19.4)
OB	1	-	3.9 (2.9–5.4)	1	-	4.0 (2.6–6.3)	1	-	3.8 (2.4–6.1)
**Estonia**									
OW/OB	1	-	8.3 (6.6–10.5)	1	-	9.3 (6.8–12.7)	1	-	7.6 (5.4–10.7)
OB	1	-	1.8 (1.1–3.1)	1	-	2.4 (1.3–4.5)	1	-	1.3 (0.5–3.0)
**Finland**									
OW/OB	1	-	12.0 (10.5–13.7)	-	-	-	-	-	-
OB	-	-	-	-	-	-	-	-	-
**France**									
OW/OB	2	75.6	11.0 (7.7–15.4)	-	-	-	-	-	-
OB	2	0.0	2.3 (1.8–2.7)	-	-	-	-	-	-
**Germany**									
OW/OB	1	-	12.7 (10.9–14.8)	1	-	10.9 (8.6–13.8)	1	-	14.5 (11.9–17.6)
OB	1	-	3.2 (2.3–4.4)	1	-	4.1 (2.8–6.1)	1	-	2.5 (1.5–4.1)
2006–2016									
	All			Girls			Boys		
Country	Number of studies	I^2	Pooled estimated (95 % CI)	Number of studies	I^2	Pooled estimated (95 % CI)	Number of studies	I^2	Pooled estimated (95 % CI)
**Greece**									
OW/OB	3	98.6	29.6 (14.5–45.0)	3	96.7	30.6 (15.7–45.5)	3	98.0	28.5 (12.9–44.1)
OB	2	99.0	8.1 (1.3–15.0)	2	98.2	8.6 (1.2–16.0)	2	97.8	7.8 (1.4–14.2)
**Hungary**									
OW/OB	1	-	11.9 (10.3–13.8)	1	-	13.0 (10.7–15.7)	1	-	10.9 (8.7–13.4)

	Number of studies	I^2	Pooled estimated (95 % CI)	Number of studies	I^2	Pooled estimated (95 % CI)	Number of studies	I^2	Pooled estimated (95 % CI)
OB	1	-	4.7 (3.7–5.9)	1	-	5.8 (4.2–7.8)	1	-	3.6 (2.4–5.3)
**Ireland**									
OW/OB	2	75.1	23.9 (19.5–29.0)	2	31.7	23.2 (21.1–25.5)	2	64.3	23.1 (18.3–28.7)
OB	2	54.0	6.2 (5.4–7.2)	2	71.3	7.0 (4.1–11.5)	2	0.0	6.4 (5.2–7.8)
**Italy**									
OW/OB	3	96.4	32.4 (23.8–42.4)	3	89.3	35.4 (27.7–44.0)	3	95.1	29.1 (19.5–40.9)
OB	3	96.0	13.5 (8.1–21.4)	3	90.1	13.4 (8.4–20.7)	3	93.3	13.1 (7.6–21.8)
**Latvia**									
OW/OB	2	63.9	16.0 (14.5–17.6)	2	74.4	16.4 (13.9–19.1)	2	0.0	15.5 (14.3–16.9)
OB	2	85.4	4.4 (3.3–6.0)	2	88.4	4.1 (2.5–6.8)	2	13.3	4.9 (4.2–5.8)
**Lithuania**									
OW/OB	1	-	16.1 (14.9–17.4)	1	-	16.2 (14.5–18.0)	1	-	16.1 (14.4–17.9)
OB	1	-	5.1 (4.4–5.9)	1	-	5.1 (4.1–6.3)	1	-	5.1 (4.1–6.2)
**Malta**									
OW/OB	1	-	23.6 (21.8–25.4)	1	-	24.8 (22.3–27.5)	1	-	22.7 (20.3–25.3)
OB	1	-	9.7 (8.5–11.0)	1	-	10.6 (8.8–12.6)	1	-	9.0 (7.4–10.8)
**Poland**									
OW/OB	3	95.2	20.5 (11.9–33.2)	2	23.1	15.3 (14.1–16.7)	1	-	12.2 (11.0–13.5)
OB	3	47.1	4.3 (3.8–4.9)	2	0.0	4.0 (3.3–4.7)	1	-	4.4 (3.7–5.3)
**Portugal**									
OW/OB	5	87.3	26.4 (23.8–29.2)	5	87.1	29.5 (25.7–33.6)	5	67.7	22.4 (20.2–24.8)
OB	5	68.0	8.1 (7.1–9.2)	5	69.4	9.1 (7.6–10.9)	5	54.3	6.5 (6.7.2)
**Romania**									
OW/OB	1	-	20.9 (17.7–24.5)	1	-	23.4 (18.8–28.8)	1	-	18.0 (13.9–22.9)
OB	1	-	6.1 (4.4–8.5)	1	-	7.2 (4.7–10.9)	1	-	5.3 (3.3–8.8)
**2006–2016**									
	**All**			**Girls**			**Boys**		
	**Number of studies**	***I*²**	**Pooled estimated (95 % CI)**	**Number of studies**	***I*²**	**Pooled estimated (95 % CI)**	**Number of studies**	***I*²**	**Pooled estimated (95 % CI)**
**Serbia**									
OW/OB	1	-	23.8 (21.0–26.8)	1	-	23.0 (19.3–27.4)	1	-	24.5 (20.6–28.9)
OB	1	-	8.4 (6.7–10.5)	1	-	7.1 (5.0–10.0)	1	-	9.7 (7.1–12.9)
**Slovenia**									
OW/OB	2	76.2	18.2 (16.4–20.2)	2	0.0	18.8 (17.5–20.1)	2	74.4	17.9 (16.6–19.2)
OB	2	48.8	5.9 (5.4–6.5)	2	38.4	6.2 (5.4–7.0)	2	9.2	5.6 (4.8–6.4)
**Spain**									
OW/OB	5	94.5	22.6 (18.7–27.0)	4	92.5	24.3 (17.7–32.3)	4	80.6	22.4 (18.3–27.1)
OB	5	93.2	7.9 (5.8–10.7)	4	62.3	8.9 (7.8–10.2)	4	76.7	8.5 (6.2–11.6)
**Sweden**									

OW/OB	2	94.6	13.3 (7.3–22.8)	2	93.7	16.4 (8.1–30.4)	2	73.7	10.4 (6.7–15.6)
OB	2	84.7	2.6 (1.9–3.6)	2	37.8	2.9 (1.0–8.4)	2	0.0	2.0 (1.2–3.3)
**Switzerland**									
OW/OB	2	69.6	13.4 (12.5–14.3)	2	55.3	15.0 (13.8–16.3)	2	0.0	11.6 (10.5–12.8)
OB	2	0.0	3.1 (2.7–3.6)	2	0.0	3.4 (2.8–4.1)	2	0.0	2.9 (2.3–3.6)
**The Netherlands**									
OW/OB	2	51.4	9.7 (9.0–10.4)	2	34.8	11.0 (10.1–12.1)	2	69.2	7.1 (5.7–8.8)
OB	2	94.9	1.5 (0.6–2.4)	2	96.7	1.6 (0.3–2.9)	2	0.0	1.2 (0.9–1.6)
**Turkey**									
OW/OB	1	-	15.5 (12.4–19.0)	1	-	15.0 (10.9–20.2)	1	-	15.8 (11.8–21.1)
OB	1	-	2.8 (1.6–4.7)	1	-	1.8 (0.7–4.6)	1	-	3.8 (2.0–7.1)
**United Kingdom**									
OW/OB	1	-	24.0 (20.2–28.3)	1	-	24.2 (19.0–30.5)	1	-	23.7 (18.4–29.9)
OB	1	-	6.4 (4.4–9.1)	1	-	5.6 (3.2–9.6)	1	-	7.1 (4.3–11.5)
**Yugoslav Republic of Macedonia**									
OW/OB	1	-	24.5 (22.9–26.1)	1	-	22.9 (20.7–25.2)	1	-	26.2 (24.0–28.5)
OB	1	-	10.2 (9.1–11.4)	1	-	9.6 (8.1–11.3)	1	-	10.9 (9.4–12.6)

- , no published article was found in the selected years.

References of included studies

1. Wijnhoven TM, van Raaij JM, Spinelli A, Rito AI, Hovengen R, Kunesova M, et al. WHO European Childhood Obesity Surveillance Initiative 2008: weight, height and body mass index in 6-9-year-old children. Pediatr Obes. 2013; 8:79-97.

2. Ahrens W, Pigeot I, Pohlabeln H, De Henauw S, Lissner L, Molnár D, et al. Prevalence of overweight and obesity in European children below the age of 10. Int J Obes (Lond). 2014;38 Suppl 2: S99-107.

3. Wijnhoven TM, van Raaij JM, Spinelli A, Starc G, Hassapidou M, Spiroski I, et al. WHO European Childhood Obesity Surveillance Initiative: body mass index and level of overweight among 6-9-year-old children from school year 2007/2008 to school year 2009/2010. BMC Public Health. 2014; 14:806.

4. Ahrens W, Siani A, Adan R, De Henauw S, Eiben G, Gwozdz W, et al. Cohort Profile: The transition from childhood to adolescence in European children-how I.Family extends the IDEFICS cohort. Int J Epidemiol. 2017;46(5):1394-1395j.

5. Lehto R, Mäki P, Ray C, Laatikainen T, Roos E. Childcare use and overweight in Finland: cross-sectional and retrospective associations among 3- and 5-year-old children. Pediatr Obes. 2016;11(2):136-43.

6. Lioret S, Touvier M, Dubuisson C, Dufour A, Calamassi-Tran G, Lafay L, et al. Trends in child overweight rates and energy intake in France from 1999 to 2007: relationships with socioeconomic status. Obesity (Silver Spring). 2009 May;17(5):1092-100.

7. Thibault H, Carriere C, Langevin C, Kossi Déti E, Barberger-Gateau P, Maurice S. Prevalence and factors associated with overweight and obesity in French primary-school children. Public Health Nutr. 2013; 16:193-201.

8. Smpokos EA, Linardakis M, Papadaki A, Kafatos A. Secular changes in anthropometric measurements and blood pressure in children of Crete, Greece, during 1992/93 and 2006/07. Prev Med. 2011 Mar-Apr;52(3-4):213-7.

9. Kleanthous K, Dermitzaki E1, Papadimitriou DT1, Papaevangelou V1, Papadimitriou A. Overweight and obesity decreased in Greek schoolchildren from 2009 to 2012 during the early phase of the economic crisis. Acta Paediatr. 2016; 105:200-5

10. Pikramenou V, Dimitraki D, Zoumpoulakis M, Verykouki E, Kotsanos N. Association between dental caries and body mass in preschool children. Eur Arch Paediatr Dent. 2016; 17:171-5.

11. Barron C, Comiskey C, Saris J. Prevalence rates and comparisons of obesity levels in Ireland. Br J Nurs. 2009; 18:799-803.

12. Toselli S, Zaccagni L, Celenza F, Albertini A, Gualdi-Russo E. Risk factors of overweight and obesity among preschool children with different ethnic background. Endocrine. 2015 Aug;49(3):717-25.

13. Bac A, Woźniacka R, Matusik S, Golec J, Golec E. Prevalence of overweight and obesity in children aged 6-13 years-alarming increase in obesity in Cracow, Poland. Eur J Pediatr. 2012 Feb;171(2):245-51.

14. Kowal M, Kryst Ł, Woronkowicz A, Sobiecki J. Long-term changes in body composition and prevalence of overweight and obesity in girls (aged 3-18 years) from Kraków (Poland) from 1983, 2000 and 2010. Ann Hum Biol. 2014; 4:415-27.

15. Kułaga Z, Gurzkowska B, Grajda A, Wojtyło M, Góźdź M, Litwin M. The prevalence of overweight and obesity among Polish pre-school-aged children. Dev Period Med. 2016; 20:143-9.

16. Merkiel S, Chalcarz W. Preschool diets in children from Piła, Poland, require urgent intervention as implied by high risk of nutrient inadequacies. J Health Popul Nutr. 2016 Apr 19;35:11.

17. Bingham DD, Varela-Silva MI, Ferrão MM, Augusta G, Mourão MI, Nogueira H, et al. Socio-demographic and behavioral risk factors associated with the high prevalence of overweight and obesity in Portuguese children. Am J Hum Biol. 2013; 25:733-42.

18. Vale S, Trost S, Ruiz JJ, Rêgo C, Moreira P, Mota J. Physical activity guidelines and preschooler's obesity status. Int J Obes (Lond). 2013 Oct;37(10):1352-5.

19. Ministério da Saúde, Instituto Nacional de Saúde Doutor Ricardo Jorge, e outro. Childhood Obesity Surveillance Initiative: COSI Portugal 2013/ Instituto Nacional de Saúde Doutor Ricardo Jorge, Direcção-Geral da Saúde; Rito A, Graça P – Lisboa, IP 2015.Link:http://repositorio.insa.pt/bitstream/10400.18/3108/3/Relatorio_COSI_Portugal_2013.pdf

20. Silva-Santos S, Santos A1, Vale S1, Mota J. Motor fitness and preschooler children obesity status. J Sports Sci. 2017; 35:1704-1708.

21. Barbu CG, Teleman MD, Albu AI3, Sirbu AE, Martin SC, Bancescu A, et al. Obesity and eating behaviors in school children and adolescents -data from a cross sectional study from Bucharest, Romania. BMC Public Health. 2015; 15:206.

22. Djordjic V, Radisavljevic S, Milanovic I, Bozic P, Grbic M, Jorga J, et al. WHO European Childhood Obesity Surveillance Initiative in Serbia: a prevalence of overweight and obesity among 6-9-year-old school children. J Pediatr Endocrinol Metab. 2016; 29:1025-30.

23. García García E, Vázquez López MÁ, Galera Martínez R, Alias I, Martín González M, Bonillo Perales A, et al. Prevalence of overweight and obesity in children and adolescents aged 2-16 years. Endocrinol Nutr. 2013; 60:121-6.

24. González García A, Álvarez Bueno C, Lucas de la Cruz L, Sánchez López M, Solera Martínez M, Díez Fernández A, et al. [Prevalence of thinness, overweight and obesity among 4-to-6-year-old spanish schoolchildren in 2013; situation in the European context]. Nutr Hosp. 2015 Oct 1;32(4):1476-82. doi: 10.3305/nh.2015.32.4.9508.

25. Gómez Santos SF, Estévez Santiago R, Palacios Gil-Antuñano N, Leis Trabazo MR, Tojo Sierra R, Cuadrado Vives C5, et al. THAO-CHILD health programme: community based intervention for healthy lifestyles promotion to children and families: results of a cohort study. Nutr Hosp. 2015;32:2584-7.

26. Garmy P, Clausson EK, Nyberg P, Jakobsson U. Overweight and television and computer habits in Swedish school-age children and adolescents: a cross-sectional study. Nurs Health Sci. 2014 Jun;16(2):143-8.

27. Jeannot E, Mahler P, Duperrex O, Chastonay P. Evolution of overweight and obesity among elementary school children in Geneva. Swiss Med Wkly. 2010 Jul 22;140:w13040.

28. Jeannot E, Mahler P, Elia N3, Cerruti B, Chastonnay P. Sociodemographic and Economic Determinants of Overweight and Obesity for Public-school Children in Geneva State, Switzerland: A Cross-sectional Study. Int J Prev Med. 2015; 6:39.

29. de Wilde JA, van Dommelen P, Middelkoop BJ, Verkerk PH. Trends in overweight and obesity prevalence in Dutch, Turkish, Moroccan and Surinamese South Asian children in the Netherlands. Arch Dis Child. 2009;94(10):795-800

30. Schönbeck Y, Talma H, van Dommelen P, Bakker B, Buitendijk SE, Hirasing RA, et al. Increase in prevalence of overweight in Dutch children and adolescents: a comparison of nationwide growth studies in 1980, 1997 and 2009. PLoS One. 2011;6(11):e27608.

31. Senol V, Unalan D, Bayat M, Mazicioglu MM, Ozturk A, Kurtoglu S. Change in reference body mass index percentiles and deviation in overweight and obesity over 3 years in Turkish children and adolescents. J Pediatr Endocrinol Metab. 2014; 27:1121-9.

32. Basterfield L, Jones AR, Parkinson KN, Reilly J, Pearce MS2, Reilly JJ, et al. Physical activity, diet and BMI in children aged 6-8 years: a cross-sectional analysis. BMJ Open. 2014;4: e005001.
